# Chaotic attractors that exist only in fractional-order case

**DOI:** 10.1016/j.jare.2022.03.008

**Published:** 2022-03-21

**Authors:** A.E. Matouk

**Affiliations:** Department of Mathematics, College of Science Al-Zulfi, Majmaah University, Al-Majmaah 11952, Saudi Arabia; College of Engineering, Majmaah University, Al-Majmaah 11952, Saudi Arabia

**Keywords:** Fractional-order, Integer-order, Chaos, Quasi-periodic attractor, Projective synchronization, LAS, Locally Asymptotically Stable, GAS, Globally Asymptotically Stable, ODEs, Ordinary Differential Equations, FOS, Fractional-Order Systems, IOS, Integer-Order System, Les, Lyapunov exponents

## Abstract

**Introduction:**

Studying chaotic dynamics in fractional- and integer-order dynamical systems has let researchers understand and predict the mechanisms of related non-linear phenomena.

**Objectives:**

Phase transitions between the fractional- and integer-order cases is one of the main problems that have been extensively examined by scientists, economists, and engineers. This paper reports the existence of chaotic attractors that exist only in the fractional-order case when using the specific selection of parameter values in a new hyperchaotic (Matouk’s) system.

**Methods:**

This paper discusses stability analysis of the steady-state solutions, existence of hidden chaotic attractors and self-excited chaotic attractors. The results are supported by computing basin sets of attractions, bifurcation diagrams and the Lyapunov exponent spectrum. These tools verify the existence of chaotic dynamics in the fractional-order case; however, the corresponding integer-order counterpart exhibits quasi-periodic dynamics when using the same choice of initial conditions and parameter set. Projective synchronization via non-linear controllers is also achieved between drive and response states of the hidden chaotic attractors of the fractional Matouk’s system.

**Results:**

Dynamical analysis and computer simulation results verify that the chaotic attractors exist only in the fractional-order case when using the specific selection of parameter values in the Matouk’s hyperchaotic system.

**Conclusions:**

An example of the existence of hidden and self-excited chaotic attractors that appears only in the fractional-order case is discussed. So, the obtained results give the first example that shows chaotic states are not necessarily transmitted between fractional- and integer-order dynamical systems when using a specific selection of parameter values. Chaos synchronization using the hidden attractors’ manifolds provides new challenges in chaos-based applications to technology and industrial fields.

## Highlights

• A novel fractional-order hyperchaotic system is investigated.
• Existence of chaotic attractors just in the fractional-order case, in this system, are reported.
• Bifurcation diagrams and corresponding Lyapunov spectrum are computed.
• The work reports the first example that shows existence of chaotic attractors that exist only in fractional-order case.

## Graphical abstract

**Figure:**
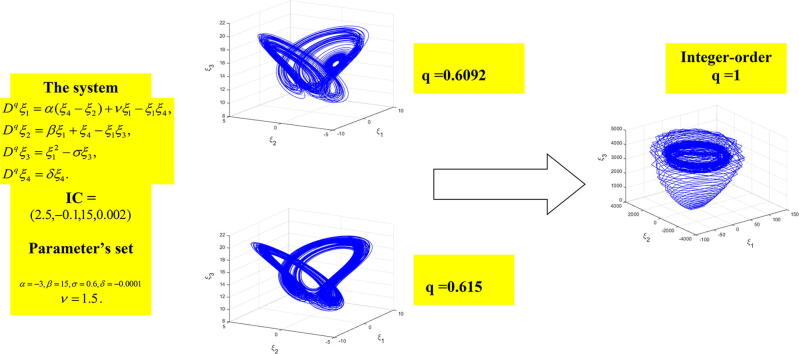

## Introduction

Fractional calculus has recently been widely explored by scientists, economists, and engineers as it provides higher adequacy and better descriptions of natural phenomena. Moreover, fractional analysis has recent developments and essential applications in science, economy and technology [1], [2], [3], [4], [5], [6], [7], [8], [9], [10], [11], [12], [13], [14], [15], [16], [17], [18], [19], [20], [21], [22], [23], [24], [25]. The equations to generate hyperchaos have received growing interest since the pioneering work by O. E. Rössler [26]. Hyperchaotic systems have been successfully applied in engineering fields and technologies [27], [28], [29], [30]. Hyperchaotic behaviors are characterized by the existence of abundant dynamics and complex behaviors, which make them better candidates in encryption algorithms [31], [32]. Recently, some systems of ODEs to generate hyperchaos have been reported by authors such as the equations by Chen [33], Lü [34], and Matouk [35]. On the other hand, investigating the dynamics of fractional-order systems (FOS) has also been considered as important research topics. Therefore, fractional-order versions of hyperchaotic systems have recently been published, such as Rössler’s [36], Chen’s [37], and Matouk’s [38] FOS.

The existence of hidden and self-excited chaotic attractors in FOS and their counterparts in the form of an integer-order system (IOS), and phase transitions of hidden attractors between the fractional- and integer-order cases are two of the main challenging problems that have been extensively examined by scientists, economists, and engineers. It was thought that if chaotic attractors exist in the IOS then they also exist in the corresponding FOS, and vice versa. This work provides a counter example to this concept. Here, chaotic attractors are shown to exist only in Matouk’s FOS when using a specific selection of parameter values and initial conditions. Moreover, projective synchronization via non-linear controllers, is also achieved between the drive and the response states of the hidden chaotic attractors of the fractional Matouk’s system that may have useful applications in some industrial, technological and military fields.

This work has promising applications in many industrial and engineering fields since the emergence of hidden chaotic attractors in the fractional-order case can be applied to chaos anti-control (chaotification) of dynamical systems which give rise to many useful chaotic signals. Therefore, such interesting phenomena can be used in chaos-based applications used in industry and technology such as path planning, image secure communication and encryption algorithms. The later application can be implemented based on the fractional Matouk’s system where the fractional parameter can be used as the secret keys of the system. Furthermore, the generation of chaos in some dynamical systems helps to produce excellent chaotic systems that can be used in improving the effect of some industrial applications such as fluid mixing.

Finally, the organization of this paper can be summarized as section (1) introduction; (2) Methods used in this work including some basic concepts of fractional analysis; (3) The main results that reports the basic contributions of this work including the interesting foundation on existence of the hidden chaotic attractors only in the fractional-order case of the Matouk’s system and the existence of self-excited chaotic attractors; (4) Projective synchronization; and (5) Conclusion.

## Methods

Here we use the preliminaries of fractional calculus to describe the systems in this work. The Caputo fractional differential operator [39] can be expressed as.

(1) $${\;}^{C}D_{t_{0}}^{q}\eta \left(t\right)=\left(\int_{t_{0}}^{t}{\left(t-\varsigma \right)}^{n-q-1}\eta^{\left(n\right)}\left(\varsigma \right)d\varsigma \right)/\Gamma \left(n-q\right),\;\;\;\;\;\;\;t>0,$$

where $q\in R^{+}$ provided that q lies inside the interval (n-1,n), and n∈N. The symbol Γ(.) represents the well-known function of Euler’s Gamma and $\eta^{\left(n\right)}\left(\varsigma \right)$ represents $\frac{d^{n}\eta \left(\varsigma \right)}{d\varsigma^{n}}.$ The operator ${\;}^{C}D_{t_{0}}^{q}$ can be rewritten as ${\;}^{C}D^{q}$ when $t_{0}=0.$ Furthermore, the Caputo type of fractional derivatives can be rewritten as.

(2) $${\;}^{C}D^{q}\;\eta \left(t\right)=J^{n-q}\eta^{\left(n\right)}\left(t\right),$$

where J is the Riemann-Liouville integral operator. Let $\overset{¯}{\Xi }\in R^{n}$ be an equilibrium state of the general n-dimensional non-linear fractional system,.

(3) $$\frac{d^{q}\Xi_{k}}{dt^{q}}=f_{k}\left(\Xi \right),\;\;\;0<q⩽1\;,\;\;k=1,\;...,\;n,$$

f is non-linear vector function. Define an arbitrary element of the Jacobian matrix $A(\overset{¯}{\Xi })$ of system (3) as $a_{\mathrm{kj}}={\left(\frac{\partial f_{k}}{\partial \Xi_{j}}\right|}_{\overset{¯}{\Xi }},\;\;\;j=1,\;...,\;n,$ then the local stability of $\overset{¯}{\Xi }$ is determined via the following theorem:

Theorem 1 **([6], [40])**

*The equilibrium point*$\overset{¯}{\Xi }$*of the system*(3)*is LAS if all the eigenvalues*$\gamma_{k}$*of the matrix*$A(\overset{¯}{\Xi })$*satisfy*.

(4) $$q<\frac{2}{\pi }\left|\arg(\gamma_{k})\right|,\;k=1,\;\;...\;.\;\;n,$$

Another basic lemma for the stability of fractional-order system (3) is also given as.

Lemma 1 **([41])**

*Assume that*Ξ:t→R*defines continuous and differentiable function. Let*$t^{*}$*be a specific instant of time, then for any*$t⩾t^{*}$*and*0<q<1,*then*.

$$0.5{\;}^{C}D_{t^{*}}^{q}\Xi^{2}\left(t\right)⩽\Xi \left(t\right){\;}^{C}D_{t^{*}}^{q}\Xi \left(t\right).$$

A new hyperchaotic system as introduced by Matouk [35,38] is given as.

(5) $$\begin{array}{c} {\;}^{C}D^{q}\;\xi_{1}=\alpha \left(\xi_{4}-\xi_{2}\right)+\nu \xi_{1}-\xi_{1}\xi_{4},\\ {\;}^{C}D^{q}\;\xi_{2}=\beta \xi_{1}+\xi_{4}-\xi_{1}\xi_{3},\\ {\;}^{C}D^{q}\;\xi_{3}=\xi_{1}^{2}-\sigma \xi_{3},\\ {\;}^{C}D^{q}\;\xi_{4}=\delta \xi_{4}. \end{array}$$

where ${\;}^{C}D^{q}$ refers to the Caputo-type fractional differential operator with an initial time of zero, 0<q⩽1, and, α,β,δ,ν,σ∈R. The equilibrium states of the system in Eq. (5) are.

(6) $$\Xi^{\left(0\right)}=\left(0,0,0,0\right),\;\;\Xi^{\left(1\right)}=\left(\sqrt{\beta \sigma },\frac{\nu \sqrt{\beta \sigma }}{\alpha },\beta ,0\right),\;\;\Xi^{\left(2\right)}=\left(-\sqrt{\beta \sigma },-\frac{\nu \sqrt{\beta \sigma }}{\alpha },\beta ,0\right).$$

Equation (5) has the Jacobian matrix of.

(7) $$A=\left(\begin{array}{cccc} -\xi_{4}+\nu & -\alpha & 0 & \alpha -\xi_{1}\\ \beta -\xi_{3} & 0 & -\xi_{1} & 1\\ 2\xi_{1} & 0 & -\sigma & 0\\ 0 & 0 & 0 & \delta \end{array}\right).$$

The matrix $A(\Xi^{\left(0\right)})$ has the eigenvalues of.

(8) $$\gamma_{1}=-\sigma ,\;\;\;\gamma_{2}=\delta ,\;\;\;\gamma_{3,4}=\frac{\nu \pm \sqrt{\nu^{2}-4\alpha \beta }}{2}.$$

For q=1, the necessary and sufficient condition for point $\Xi^{\left(0\right)}$ to be LAS is.

(9) $$\alpha \beta \in R^{+},\;\;\sigma \in R^{+},\;\;\delta \in R^{-},\;\;\;\;\nu \in R^{-}.$$

The eigenvalue equation of points $\Xi^{\left(1\right)}$ and $\Xi^{\left(2\right)}$ is.

(10) $$\gamma^{4}+\left(\sigma -\delta -\nu \right)\gamma^{3}+\left(\nu \delta -\nu \sigma -\sigma \delta \right)\gamma^{2}+\left(\nu \sigma \delta -2\alpha \beta \sigma \right)\gamma +2\alpha \beta \sigma \delta =0.$$

The local stability of these equilibrium states when q∈(0,1) is investigated in [38].

Finally, this section is ended by the following basic definition [42].

Definition 1 Consider the general system (3). If there exists an attractor T such that its basin of attraction intersects with any open neighborhood of $\overset{¯}{\Xi }$ then T is said to be a self-excited attractor, otherwise, T is called a hidden attractor.

## The main results

The fractional system in Eq. (5) is numerically integrated based on the predictor–corrector algorithm [43], [44], [45] with a step size of h = 0.005, using the parameter values α=-3,β=15,σ=0.6,δ=-0.0001 with ν=1.5 and initial conditions of${\left(2.5,-0.1,15,0.002\right)}^{\text{T}}$. Hence, the equilibria defined by Eq. (6) are written as.

(11) $$\Xi^{\left(0\right)}=\left(0,0,0,0\right),\;\;\Xi^{\left(1\right)}=\left(3,-1.5,15,0\right),\;\;\Xi^{\left(2\right)}=\left(-3,1.5,15,0\right).$$

The eigenvalues of the matrix A defined in Eq. (7) evaluated at $\Xi^{\left(0\right)}$ have the form given in Eq. (8). So, the eigenvalues in Eq. (8), with the above-mentioned selection of parameter values, are reduced to.

$$\gamma_{1}=-0.6,\;\gamma_{2}=-0.0001,\;\gamma_{3}=7.5,\;\gamma_{4}=-6,$$

which implies that $\Xi^{\left(0\right)}$ is a saddle point for allq∈(0,1]. Obviously, if the Eq. (10) is divided by the factor(γ-δ), then the quotient is given by the polynomial.

(12) $$P\left(\gamma \right)=\gamma^{3}+\left(\sigma -\nu \right)\gamma^{2}-\nu \sigma \gamma -2\alpha \beta \sigma .$$

Hence, the eigenvalues ($\gamma_{k},\;\;k=1,2,3,4$) of the Jacobian A evaluated at $\Xi^{\left(1\right)}\left(\;\Xi^{\left(2\right)}\right)$ can be determined from the roots of P(γ) and the equation γ=δ that are numerically computed as.

(13) $$\gamma_{1}=-0.0001,\;\gamma_{2}=-3.575551534,\;\gamma_{3,4}=2.237775767\pm 3.177251134i,\;\;\;\;i=\sqrt{-1}.$$

Therefore, the stability conditions in Eq. (4) indicate that the points $\Xi^{\left(1\right)}$ and $\Xi^{\left(2\right)}$ are LAS if and only ifq<0.6093611978..

**Fig. 1:**
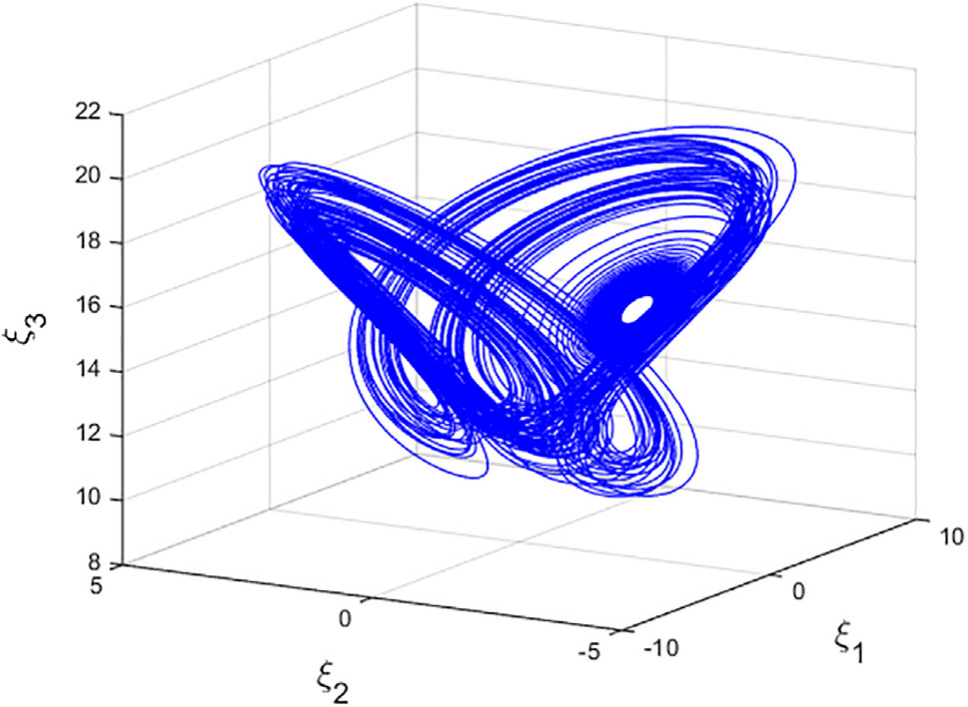
Chaotic attractor of Eq. (5) with initial conditions ${\left(2.5,-0.1,15,0.002\right)}^{T},$α=-3,β=15,σ=0.6,δ=-0.0001, ν=1.5, and q=0.6092.

**Fig. 2:**
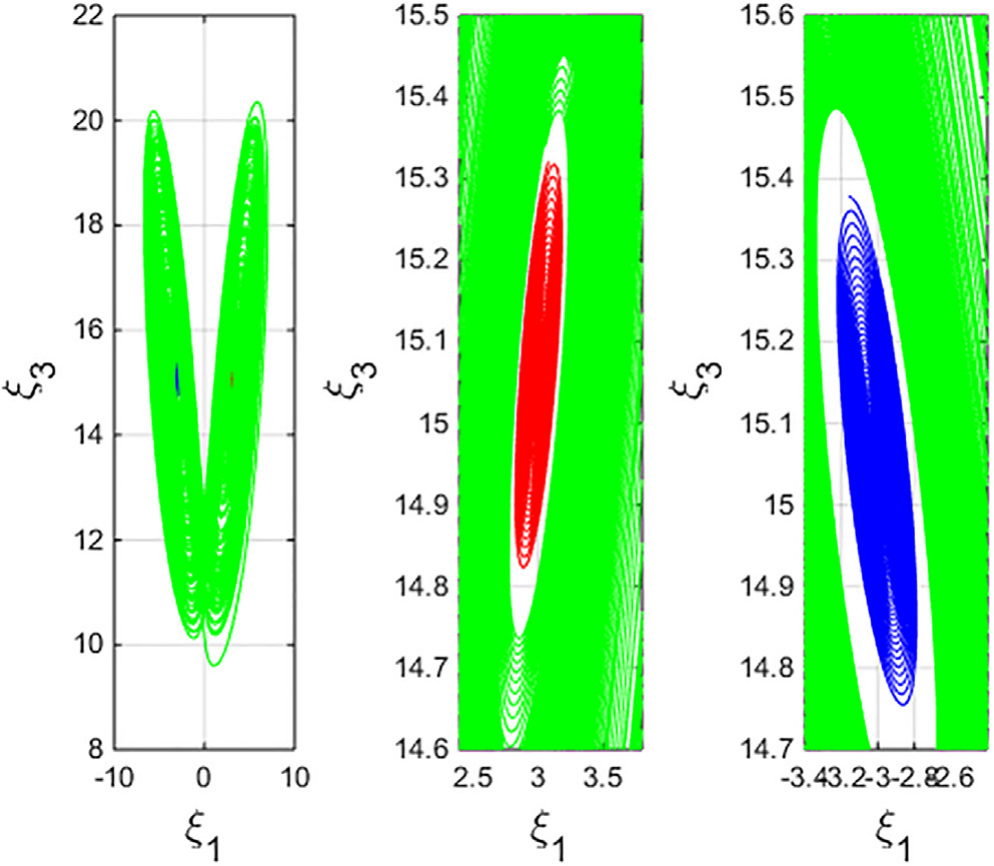
$\xi_{1}\xi_{3}-$ View of system (5) with the parameter values α=-3,β=15,σ=0.6,δ=-0.0001, ν=1.5 and q=0.6092 showing hidden chaotic attractor (green plot), one-point attractor related to $\Xi^{\left(1\right)}$ (red domain) and one-point attractor related to $\Xi^{\left(2\right)}$ (blue domain). The initial data ${\left(2.5,-0.1,15,0.002\right)}^{T},$${\left(3,1,15,0.0199\right)}^{T},$${\left(-3,-1,15,0.0199\right)}^{T}$ are selected for the green, red and blue domains, respectively.

**Fig. 3:**
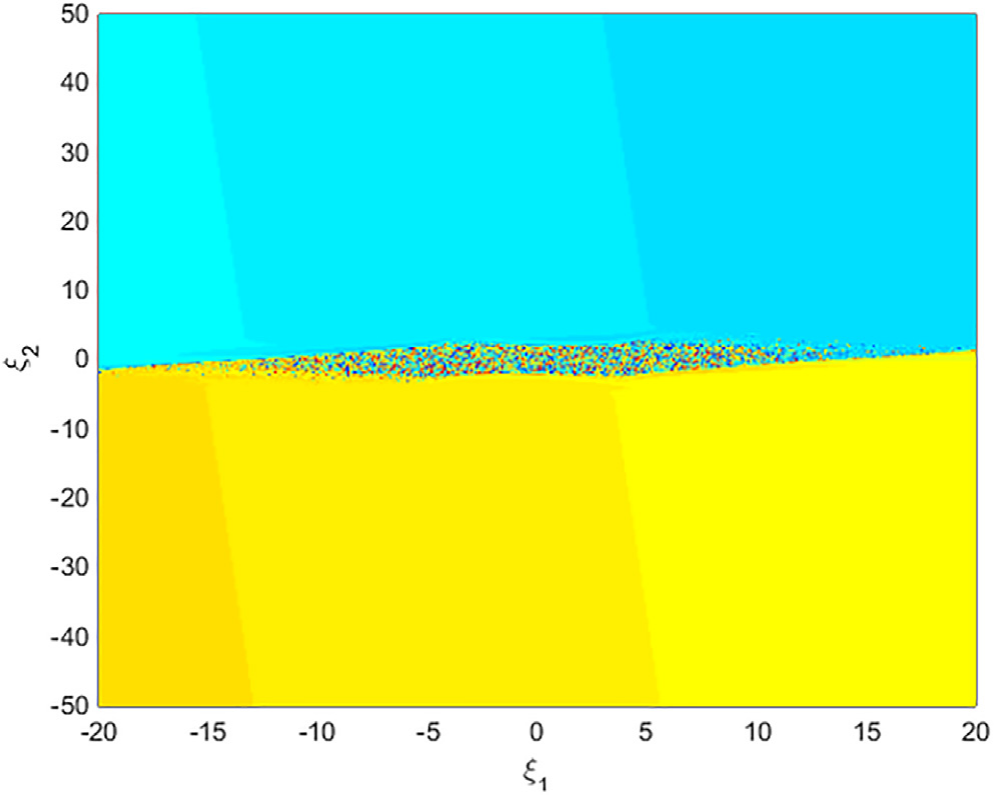
A basin set of attraction for system (5), as a cross-section in $\xi_{1}\xi_{2}-$ plane for $\xi_{3}=15,\;\xi_{4}=0.002$, showing the hidden chaotic attractors (riddled domain) with α=-3,β=15,σ=0.6,δ=-0.0001, ν=1.5, and q=0.6092.

**Fig. 4:**
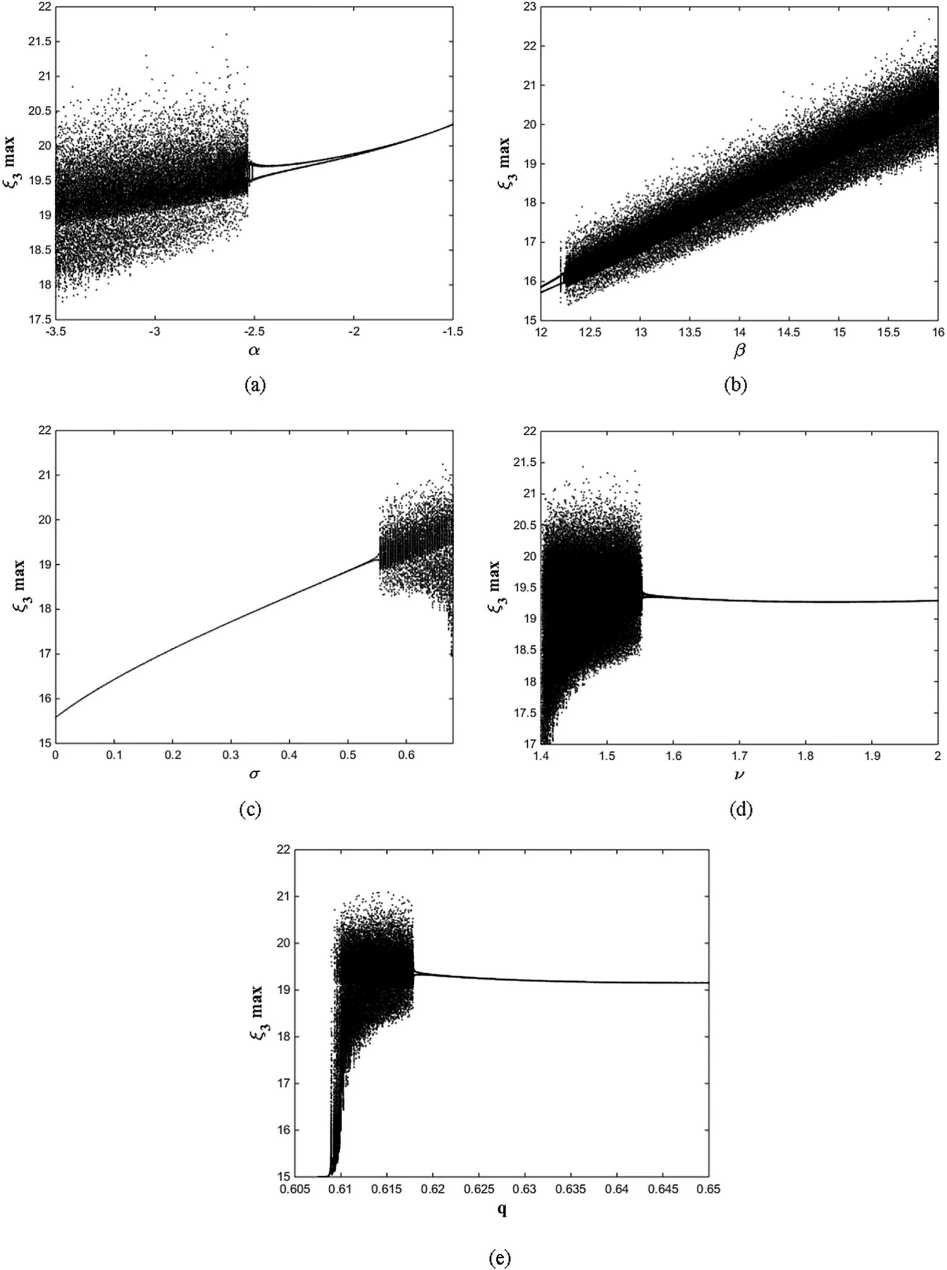
Bifurcation diagrams of Eq. (5) as (a) α varies; (b) β varies; (c) σ varies; (d) ν varies, and (e) q varies. The fixed parameter values are at α=-3,β=15,σ=0.6,δ=-0.0001, ν=1.5, and q=0.615..

**Fig. 5:**
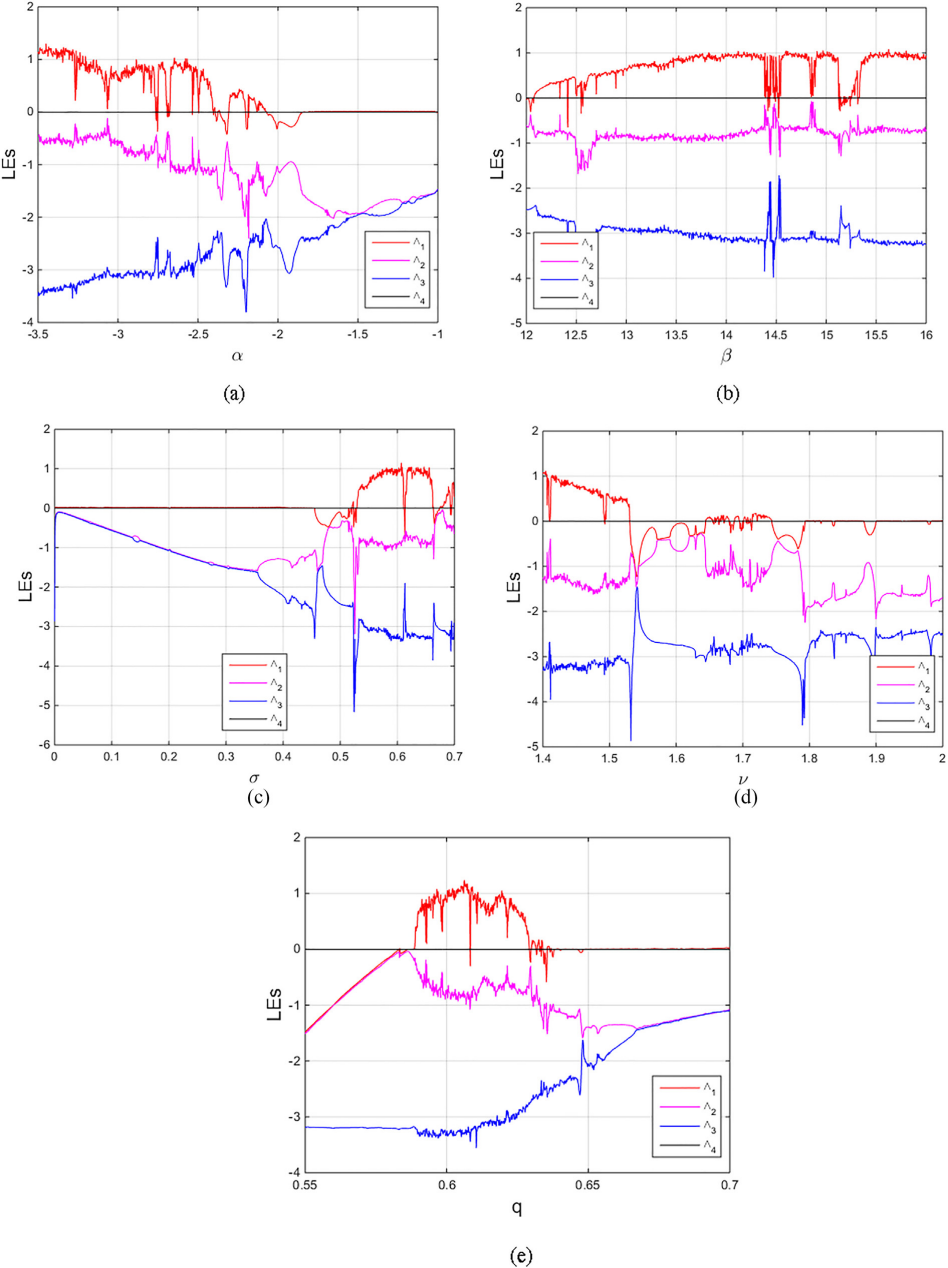
Lyapunov exponent spectra of Eq. (5) as (a) α varies; (b) β varies; (c) σ varies; (d) ν varies, and (e) q varies. The fixed parameter values are α=-3,β=15,σ=0.6,δ=-0.0001, ν=1.5, andq=0.615.

**Fig. 6:**
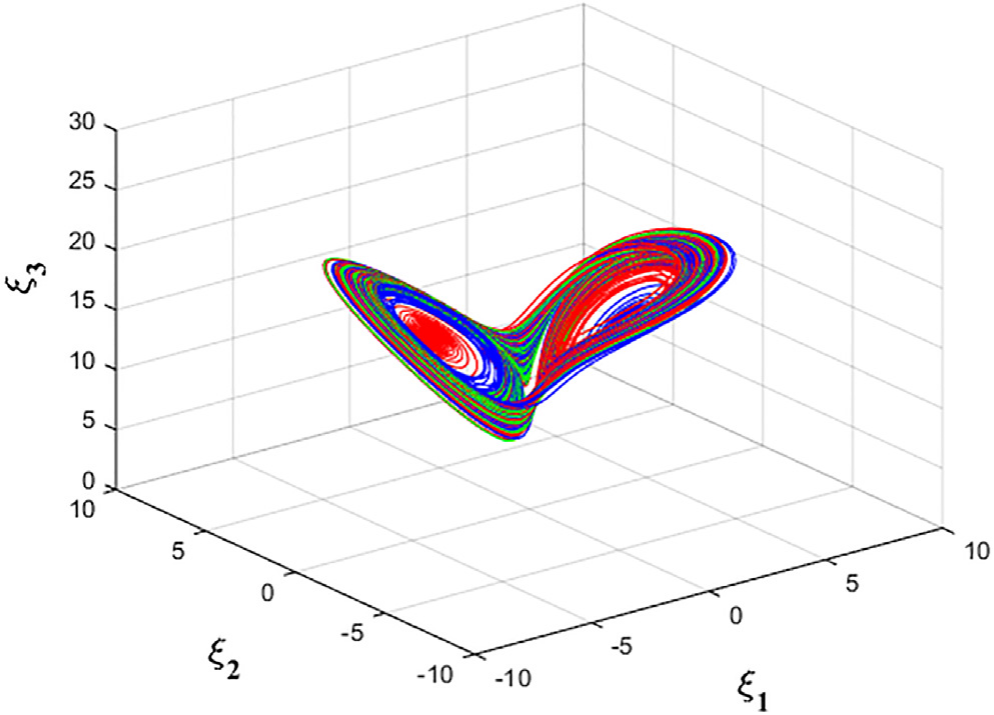
Self-excited chaotic attractor of Eq. (5) with α=-3,β=15,σ=0.6,δ=-0.0001, ν=1.5, q=0.615 and initial data ${\left(2.5,-0.1,15,0.002\right)}^{T}$ for green trajectory, ${\left(-3,1.495,15,0\right)}^{T}$ for red trajectory and ${\left(3,-1.495,15,0\right)}^{T}$ for blue trajectory.

**Fig. 7:**
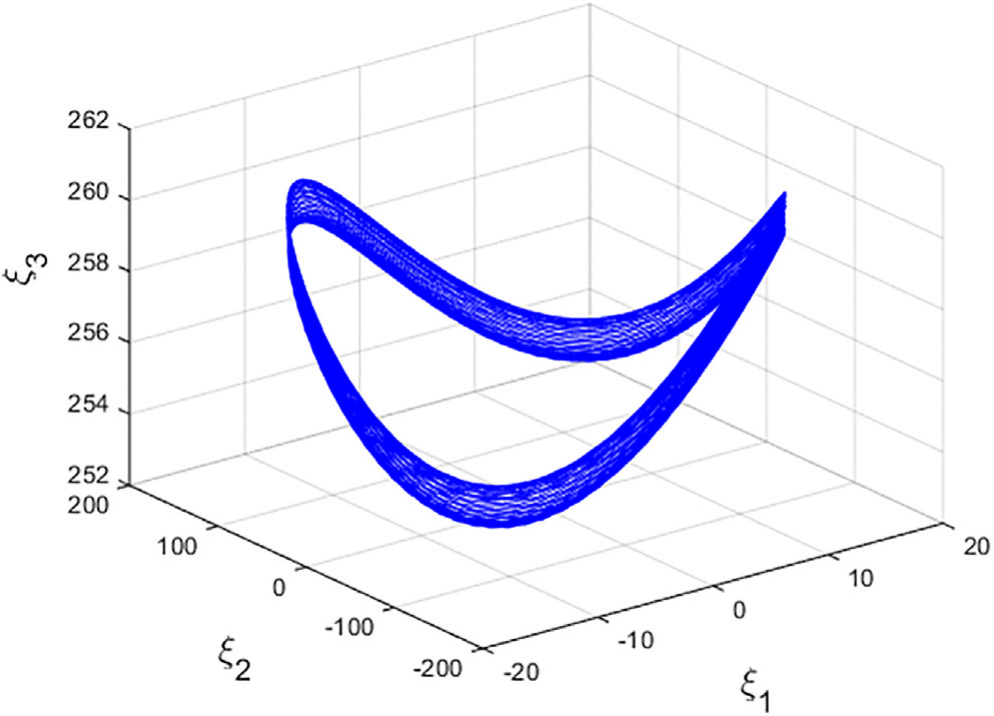
A quasi-periodic attractor of Eq. (5) with α=-3,β=15,σ=0.6,δ=-0.0001, ν=1.5 and q=0.98..

**Fig. 8:**
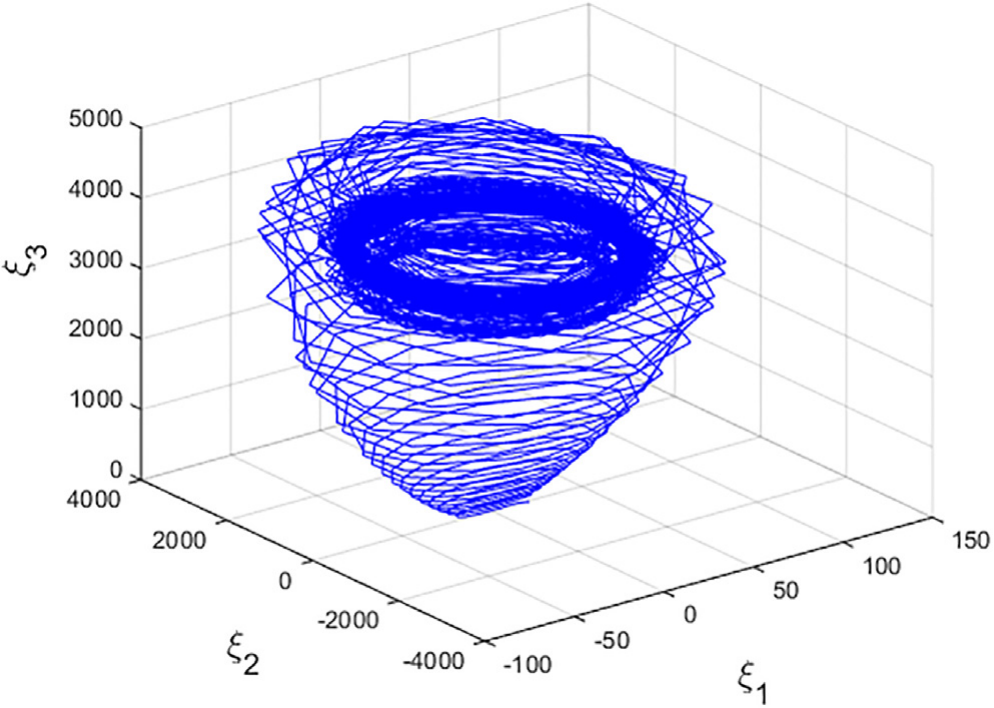
A quasi-periodic attractor of Eq. (5) with, ν=1.5, and q=1.

**Fig. 9:**
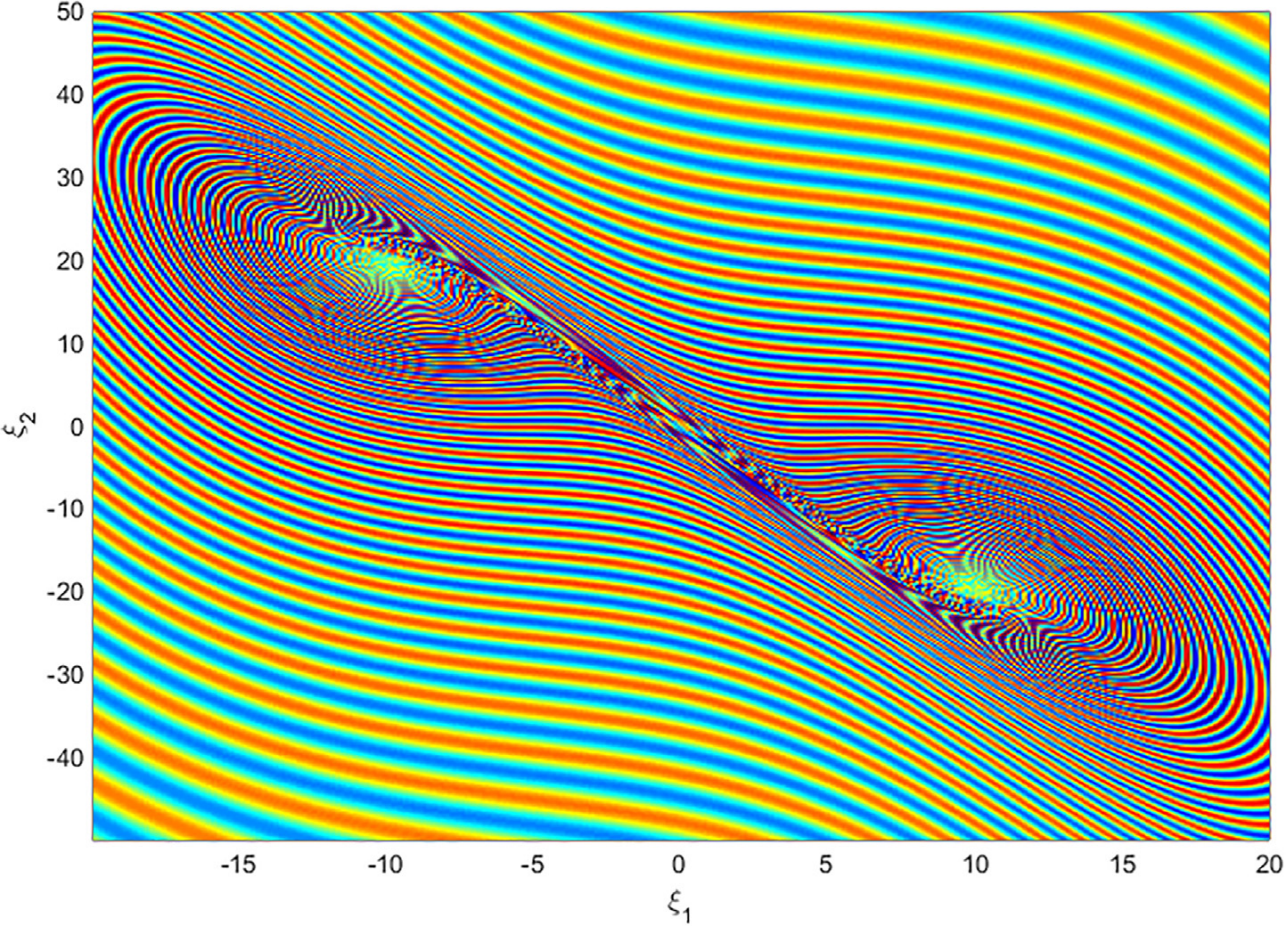
A basin set of attraction for system (5), as a cross-section in $\xi_{1}\xi_{2}-$ plane for $\xi_{3}=0,\;\xi_{4}=0$, with α=-3,β=15,σ=0.6,δ=-0.0001, ν=1.5, and q=1.

For q=0.6092 and using the above-mentioned parameter values produces the chaotic attractor, as seen in Fig. 1. Indeed, the chaotic attractor appeared in Fig. 1 is a hidden chaotic attractor according to the formal Definition 1. To discuss the existence of this hidden chaotic attractor in the fractional case of Eq. (5); the trajectories emanating in proximity to the saddle (of an index one) equilibrium state $\Xi^{\left(0\right)}$ are all attracted by the locally stable equilibrium states $\Xi^{\left(1\right)}$ and $\Xi^{\left(2\right)}$. In Fig. 2, it is shown that the trajectories originates close to the origin steady state $\Xi^{\left(0\right)}=\left(0,0,0,0\right)$ (green domain) either tend to $\;\Xi^{\left(2\right)}=\left(-3,1.5,15,0\right)$ (blue domain), or to $\Xi^{\left(1\right)}=\left(3,-1.5,15,0\right)$ (red domain) where its basins of attraction do not intersect with a certain neighborhood of the non-origin co-planar steady states. Furthermore, to verify the existence of such hidden attractors, the corresponding basin set of attraction is computed and is depicted in Fig. 3. Meanwhile, Ref. [38] indicates that an approximately periodic orbit is expected near q=0.609361198, which is broken, for a specific choice of initial data, to create the fore-mentioned hidden chaotic attractors that surround the asymptotic attractors near $\Xi^{\left(1\right)}$ and $\Xi^{\left(2\right)}$. This scenario is illustrated in the bifurcation diagrams shown in Fig. 4, which indicates that chaotic attractors exist in the fractional-order case when q∈[0.6092,0.6188). Calculations of the Lyapunov exponents (LEs or $\Lambda_{i,s}$) are performed based on the algorithm in [46]. Using these parameter values, the orders of q=0.6092 and q=0.615 give values of $\Lambda_{1}\approx 1.09,\;\;\Lambda_{2}\approx 0.00,\;\;\Lambda_{3}\approx -0.92,\;\;\Lambda_{4}\approx -3.29$ and $\Lambda_{1}\approx 0.86,\;\;\Lambda_{2}\approx 0.00,\;\;\Lambda_{3}\approx -0.69,\;\;\Lambda_{4}\approx -3.14,$ respectively. The calculations for the corresponding LEs spectra are depicted in Fig. 5. Fig. 6 shows existence of self-excited chaotic attractor when q=0.615. However, when 0.634⩽q⩽0.96, these chaotic attractors disappear and are replaced by invariant closed curves. Quasi-periodic attractors appear when q becomes sufficiently close to one. Fig. 7 shows a quasi-periodic attractor for q=0.98. When q=1, the equilibrium states $\Xi^{\left(1\right)}$ and $\Xi^{\left(2\right)}$ change their stability to become saddle foci with an index two and an unstable periodic orbit due to Hopf bifurcation appears near h=-9.191575212 and h=9.791575212 [35]. Fig. 8 shows a quasi-periodic attractor when q=1. The calculations for the integer-order form of Eq. (5) are based on an algorithm of the fourth-order Runge Kutta scheme. The corresponding basin set of attraction is depicted in Fig. 9.

## Projective synchronization for system (5) using non-linear controllers

In the following, a projective synchronization scheme is employed to the system (5). So, the drive and response systems are, respectively, given as.

(14) $$\begin{array}{c} {\;}^{C}D^{q}\;\xi_{1}^{d}=\alpha \left(\xi_{4}^{d}-\xi_{2}^{d}\right)+\nu \xi_{1}^{d}-\xi_{1}^{d}\xi_{4}^{d},\\ {\;}^{C}D^{q}\;\xi_{2}^{d}=\beta \xi_{1}^{d}+\xi_{4}^{d}-\xi_{1}^{d}\xi_{3}^{d},\\ {\;}^{C}D^{q}\;\xi_{3}^{d}={\left(\xi_{1}^{d}\right)}^{2}-\sigma \xi_{3}^{d},\\ {\;}^{C}D^{q}\;\xi_{4}^{d}=\delta \xi_{4}^{d}, \end{array}$$

(15) $$\begin{array}{c} {\;}^{C}D^{q}\;\xi_{1}^{r}=\alpha \left(\xi_{4}^{r}-\xi_{2}^{r}\right)+\nu \xi_{1}^{r}-\xi_{1}^{r}\xi_{4}^{r}+\kappa_{1}\left(t\right),\\ {\;}^{C}D^{q}\;\xi_{2}^{r}=\beta \xi_{1}^{r}+\xi_{4}^{r}-\xi_{1}^{r}\xi_{3}^{r}+\kappa_{2}\left(t\right),\\ {\;}^{C}D^{q}\;\xi_{3}^{r}={\left(\xi_{1}^{r}\right)}^{2}-\sigma \xi_{3}^{r}+\kappa_{3}\left(t\right),\\ {\;}^{C}D^{q}\;\xi_{4}^{r}=\delta \xi_{4}^{r}+\kappa_{4}\left(t\right), \end{array}$$

where $\xi_{i}^{d},\;\xi_{i}^{r},\;i=1,2,3,4$ refer to the states of drive and response systems, respectively, and $\kappa_{i}\left(t\right),\;\;i=1,2,3,4$ are non-linear controllers to be designed. Now, define the errors of synchronization as.

(16) $$\lambda_{i}=\xi_{i}^{r}-k\xi_{i}^{d},\;\;\;i=1,2,3,4,$$

where k is a scaling parameter. Also the non-linear control functions are defined as.

(17) $$\begin{array}{c} \kappa_{1}\left(t\right)=\alpha \left(\lambda_{1}+\lambda_{2}-\lambda_{4}\right)-\nu \lambda_{1}-\xi_{4}^{d}\lambda_{1}+\frac{1}{k}\left[\left(k-1\right)\xi_{1}^{r}\xi_{4}^{r}+\xi_{1}^{r}\lambda_{4}\right],\\ \kappa_{2}\left(t\right)=-\beta \left(\lambda_{1}+\lambda_{2}\right)-\lambda_{4}+\xi_{1}^{d}\lambda_{3}+\frac{1}{k}\left[\left(k-1\right)\xi_{1}^{r}\xi_{3}^{r}+\xi_{3}^{r}\lambda_{1}\right],\\ \kappa_{3}\left(t\right)=-\xi_{1}^{d}\lambda_{1}-\frac{1}{k}\left[\left(k-1\right){\left(\xi_{1}^{r}\right)}^{2}+\xi_{1}^{r}\lambda_{1}\right],\\ \kappa_{4}\left(t\right)=-\lambda_{4}. \end{array}$$

Hence, we suggest the following Lyapunov function.

(18) $$V\left(\lambda \left(t\right)\right)=\frac{1}{2}\sum_{i=1}^{4}{\left(\lambda_{i}\left(t\right)\right)}^{2},\;\;\;i=1,2,3,4.$$

According to Lemma 1, V(λ(t)) must satisfy the following inequality.

(19) $${\;}^{C}D^{q}V\left(\lambda \left(t\right)\right)⩽\sum_{i=1}^{4}\lambda_{i}{\;}^{C}D^{q}\;\lambda_{i}\left(t\right).$$

Then, after inserting the operator ${\;}^{C}D^{q}$ into the error dynamical system with the controllers (17), we obtain.

(20) $${\;}^{C}D^{q}\;\;V\left(\lambda \left(t\right)\right)⩽\alpha \lambda_{1}^{2}-\beta \lambda_{2}^{2}-\sigma \lambda_{3}^{2}+\left(\delta -1\right)\lambda_{4}^{2}.$$

If α<0,β>0,σ>0,δ<0, then ${\;}^{C}D^{q}\;\;V\left(\xi \left(t\right)\right)<0$ which implies that the origin equilibrium (stationary) state of the error dynamical system is GAS. Hence, it is shown that the synchronization between the drive and response systems is achieved under the proposed projective synchronization scheme.

**Fig. 10:**
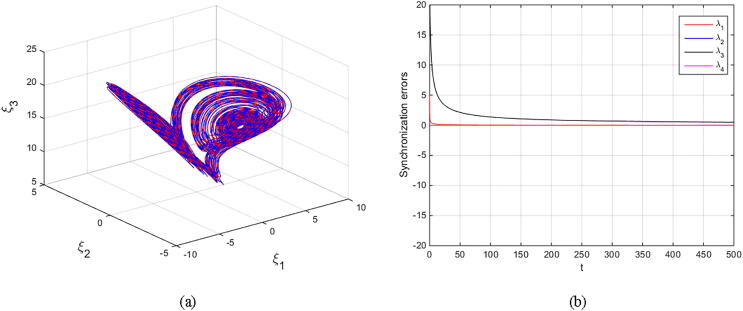
Phase space and synchronization errors between systems (14), (15) using controllers $\kappa_{i},\;i=1,2,3,4$ defined by (17) with scaling parameter k=1: (a) 3D plot of drive’s and response’s hidden chaotic attractors; (b) Corresponding synchronization errors.

**Fig. 11:**
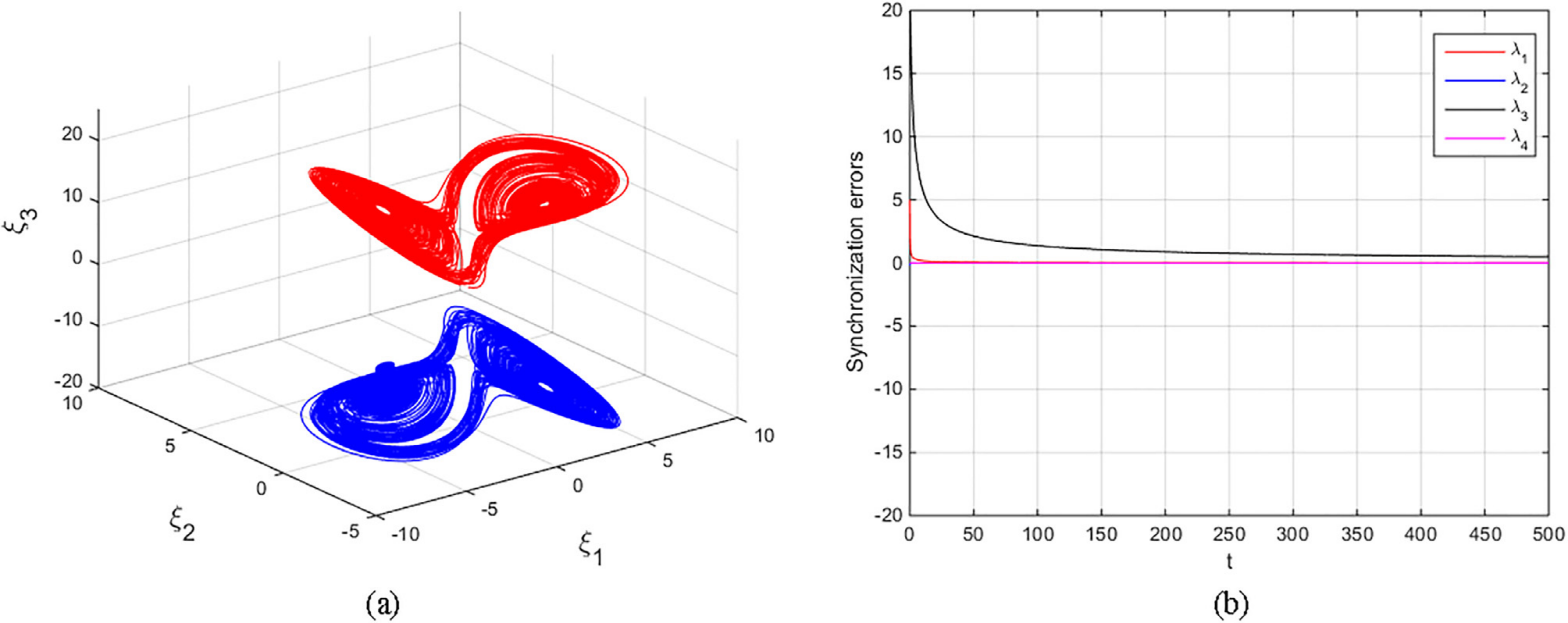
Phase space and synchronization errors between systems (14), (15) using controllers $\kappa_{i},\;i=1,2,3,4$ defined by (17) with scaling parameter k=-1: (a) 3D plot of drive’s and response’s hidden chaotic attractors; (b) Corresponding synchronization errors.

**Fig. 12:**
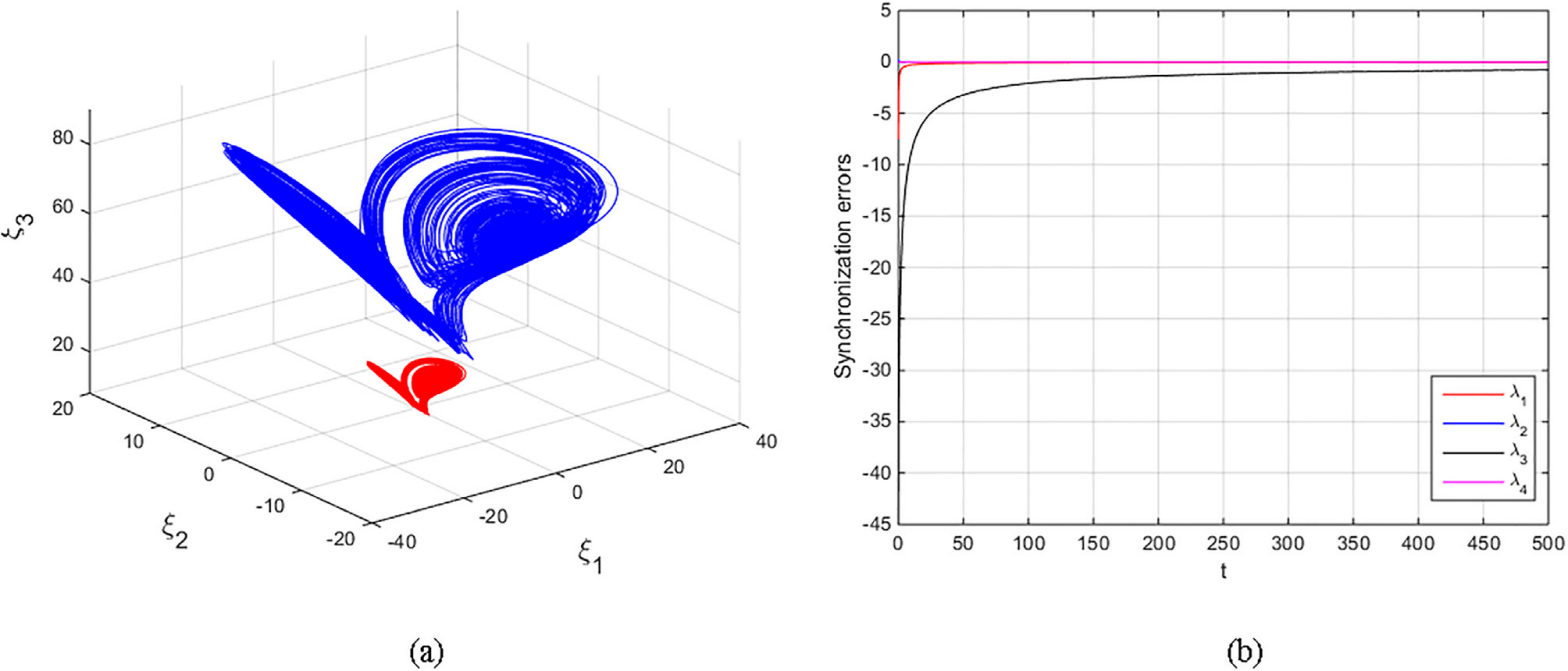
Phase space and synchronization errors between systems (14), (15) using controllers $\kappa_{i},\;i=1,2,3,4$ defined by (17) with scaling parameter k=4: (a) 3D plot of drive’s and response’s hidden chaotic attractors; (b) Corresponding synchronization errors.

**Fig. 13:**
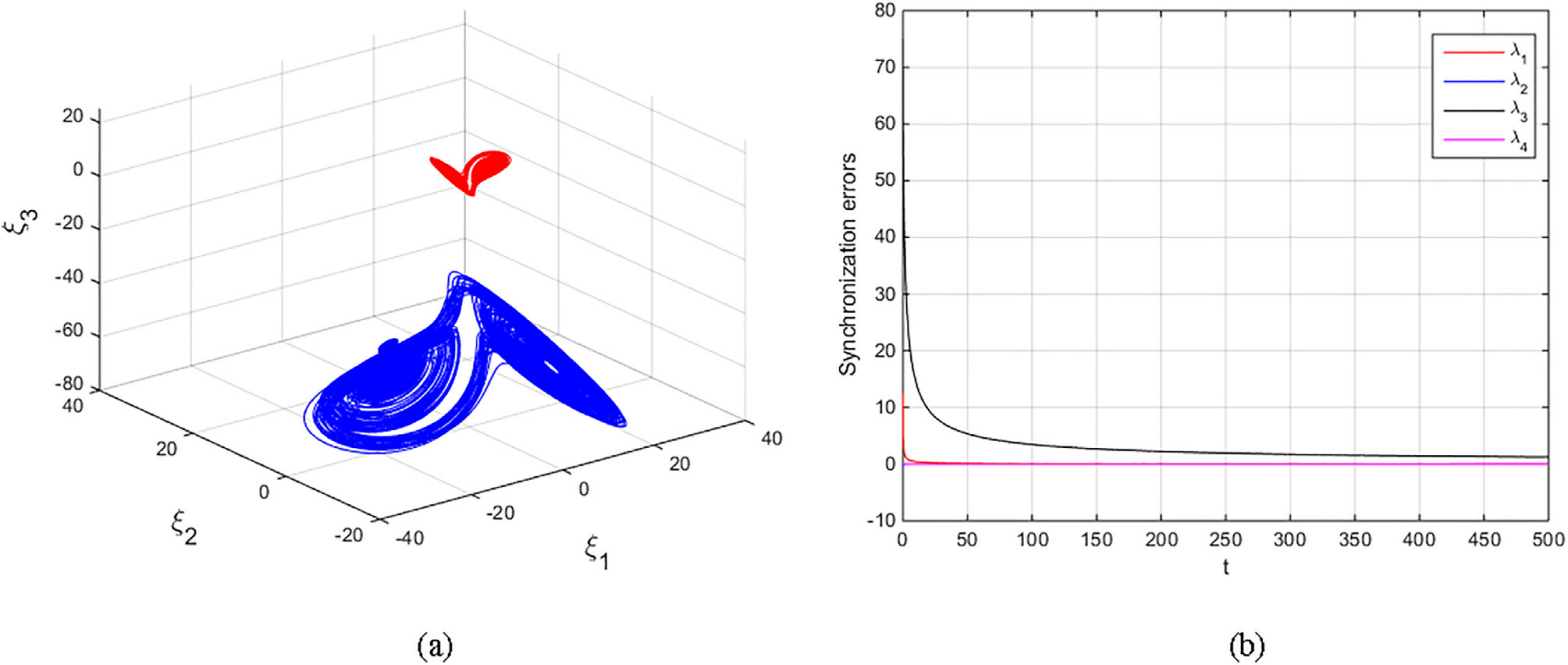
Phase space and synchronization errors between systems (14), (15) using controllers $\kappa_{i},\;i=1,2,3,4$ defined by (17) with scaling parameter k=-4: (a) 3D plot of drive’s and response’s hidden chaotic attractors; (b) Corresponding synchronization errors.

Matouk’s equations (14), (15) are numerically simulated with set α=-3,β=15,σ=0.6,δ=-0.0001,ν=1.5, and q=0.6092. The initial conditions ${\left(2.5,-0.1,15,0.002\right)}^{T}$ are selected to obtain the fore-mentioned hidden chaotic attractors. The scaling parameter is also chosen as k=±1 and k=±4. The results are depicted in Fig. 10, Fig. 11, Fig. 12, Fig. 13 in which the states of drive system have red domain and the states of response system have blue domain.

## Conclusion

To conclude this work; An example of the existence of hidden chaotic attractors in a new hyperchaotic system that appears only in the fractional-order case is discussed. The system has been shown to have three equilibrium states: the origin state and two non-origin co-planar equilibrium states. The chaotic attractors occur in the fractional case when the origin state is a saddle point and near the critical value of $q^{\left(*\right)}=0.6093611978.$ The two non-origin co-planar equilibrium states are LAS if and only if $q<q^{\left(*\right)}.$ Therefore, trajectories that begin near the origin equilibrium state converge to one of the locally stable non-origin equilibrium states, and a chaotic attractor that surrounds all these states appears. For a specific selection of initial data which have been computed via the basin sets of attractions, this scenario still takes place even when q passes the critical value of $q^{\left(*\right)}$ as the expected asymptotic periodic orbits that result from the Hopf bifurcation are broken into self-excited chaotic attractors. However, when q becomes sufficiently close or equal to one, these kinds of chaotic attractors are replaced by quasi-periodic attractors. Numerical simulations using different tools such as bifurcation diagrams, Lyapunov exponents, and strange attractors have been performed to confirm these foundations. This paper also reports the lowest order to obtain hidden chaotic attractors in four-dimensional systems (4×0.6092=2.4368). To the best of the author’s knowledge, these results have not appeared in the existing literature. Moreover, chaos projective synchronization using the hidden attractors’ manifolds has been achieved based on non-linear control theory that may provide new challenges in chaos-based applications to technology and industrial fields.

Although, this work discussed the existence of chaotic attractors in a four-dimensional system that appears only in the fractional-order case; future studies may be devoted to report similar discussion for two- and three-dimensional systems. Future works may also include hardware implementation of the proposed Matouk’s systems in addition to its circuit realization and its applications in text encryption algorithms. Investigations of the dynamics of the discretized fractional Matouk’s systems are also interesting points for research.

## Compliance with ethics requirements

This work does not contain any studies with human or animal subjects.

### CRediT authorship contribution statement

**A.E. Matouk:** Conceptualization, Methodology, Software, Data curation, Writing – original draft, Visualization, Investigation, Supervision, Software, Validation, Writing – review & editing.

## Declaration of Competing Interest

The authors declare that they have no known competing financial interests or personal relationships that could have appeared to influence the work reported in this paper.
